# Melatonin treatment of pigs with acute pancreatitis reduces inflammatory reaction of pancreatic tissue and enhances fitness score of pigs: experimental research

**DOI:** 10.1186/s13017-019-0237-2

**Published:** 2019-04-11

**Authors:** Katharina Grupp, Johannes Erbes, Annika Poppe, Karin Wodack, Andreas Gocht, Constantin Trepte, Jan Havel, Oliver Mann, Jakob R. Izbicki, Kai Bachmann

**Affiliations:** 1 Department of General, Visceral and Thoracic Surgery, University Medical Centre Hamburg-Eppendorf, Hamburg, Germany; 2 Centre of Anesthesiology and Intensive Care Medicine, University Medical Centre Hamburg-Eppendorf, Hamburg, Germany; 3 Institute of Anatomy and Experimental Morphology, University Medical Center Hamburg-Eppendorf, Hamburg, Germany

**Keywords:** Acute pancreatitis, Experimental model, Melatonin, Inflammation, Edema, Fitness score

## Abstract

**Background:**

Severe acute pancreatitis is associated with high morbidity and mortality. Melatonin is known as the activator of antioxidant enzymes. The main purpose of this study was to evaluate the clinical effect of melatonin treatment in a pig model with induced acute pancreatitis.

**Methods:**

In this study, acute pancreatitis was induced in 38 German domestic pigs (German Hybrid). After induction of acute pancreatitis, 18 animals were treated with melatonin. Intraoperative clinical data, postoperative blood parameters, fitness, and Porcine Well-being (PWB) score, and post-mortal histopathological data were analyzed in both study groups.

**Results:**

The matching procedure created two groups (melatonin group and control group) which were very similar. The fitness and PWB score were postoperative significantly enhanced in the melatonin group as compared to the control group (*p* = 0.005 and *p* = 0.003). Additionally, histological analysis revealed that acinar necrosis, fat tissue necrosis, and edema were significantly reduced in the melatonin group as compared to the non-melatonin group (*p* = 0.025, *p* = 0.003, and *p* = 0.028).

**Conclusions:**

Pigs, which were treated with melatonin, were characterized by higher fitness and PWB scores than those of the control group. Moreover, melatonin treatment reduces the acinar necrosis, fat tissue necrosis, and edema of pancreatic tissue. Thus, melatonin might be a useful therapeutic option in severe acute pancreatitis.

**Electronic supplementary material:**

The online version of this article (10.1186/s13017-019-0237-2) contains supplementary material, which is available to authorized users.

## Introduction

Acute pancreatitis is an inflammatory and potentially fatal disease [1]. The initial management of acute pancreatitis is supportive and includes optimization of electrolyte and fluid balance, providing adequate caloric support and preventing complications [2]. Local complications such as pancreatic necrosis, pancreatic pseudocysts, pancreatic duct injury, and peripancreatic vascular complications are treated by a combination of endoscopic, radiologic, and surgical techniques [2]. The surgical management for acute pancreatitis is indicated in case of acute gallstone pancreatitis or complications of acute pancreatitis [2]. Despite recent improving diagnostic and therapeutic options, acute pancreatitis is still associated with significant morbidity and mortality [1].

The hormone melatonin has been suggested to enhance the activation of antioxidant enzymes including superoxide dismutase, catalase, glutathione peroxidase, and glutathione reductase [3–5] and to reduce radical oxygen and nitrogen species [6–8]. In acute pancreatitis, melatonin has been described to play a protective role due to reduction of the gene expression and synthesis of proinflammatory cytokines such as tumor necrosis factor-α (TNFα) and proinflammatory interleukins such as interleukin (IL)-1β, IL-6, IL-8, and prostaglandins [9–12]. In addition, melatonin has been shown to modulate the processes of apoptosis and necrosis [13–15]. Moreover, studies using animal models have shown that melatonin might play a protective role in acute pancreatitis-associated organ injuries [16–19]. The main purpose of this study was to further evaluate the clinical effect of melatonin treatment in pigs after induction of acute pancreatitis.

Our data demonstrate that melatonin treatment results in a reduction of acinar necrosis, fat tissue necrosis, and edema of pancreatic tissue during acute pancreatitis. Thus, it can be speculated that melatonin might be a useful therapeutic option in the treatment of severe acute pancreatitis.

## Methods

### Animals

The study was approved by the Governmental Commission on the Care and Use of Animals of the City of Hamburg. The animals received care in compliance with the “Guide for the Care and Use of Laboratory Animals” (NIH publication No. 86-23, revised 1996). Thirty-eight of 40 pigs (German Hybrid) were included. The animals were randomized to two different treatment groups: group 1 (melatonin, *n* = 18) and group 2 (non-melatonin; control group; *n* = 20). Two of the animals died before randomization.

Based on an α error of 5% and β error of 20%, the statistical calculation resulted in at least 18 animals per groups according to Fleiss tables. The animals were randomized online using a simple web-based randomization algorithm. Acute pancreatitis was induced in both groups, but only the animals of the melatonin group were treated with melatonin. The animals were randomized online using a simple web-based randomization algorithm. After the specific treatment, the postoperative course of the animals were not blinded. But the pathologist was blinded.

### Induction of anesthesia and monitoring

The animals were randomized in two groups: group 1 (melatonin group; *n* = 18), which were treated with melatonin after induction of acute necrotizing pancreatitis, and group 2 (non-melatonin group/control group; *n* = 20). After fasting overnight with free access to water, ketamine (10 mg/kg), midazolam (0.5 mg/kg), azaperone (4 mg/kg), and atropine (0.0015 mg/kg) were administered for premedication. For monitoring of heart rate and oxygen saturation, a 5-lead electrocardiogram and pulse oximetry were used. After preoxygenation, anesthesia was induced by intravenous injection of 0.5 mg/kg midazolam. The animals were intubated and ventilated in a pressure-controlled mode assuring tidal volumes of 8–12 ml/kg and an end-expiratory pCO2 of 35–40 mmHg using an inspiratory oxygen concentration of 0.35 (Zeus, Draeger Medical Systems, Luebeck, Germany). Continuous infusion of fentanyl (0.05 mg/kg/h) and sevoflurane (Fet 2.0) was used for balanced anesthesia. After cleaning, shaving, disinfection, and sterile draping, the femoral artery was cannulated using a 5 F thermistor-tipped arterial catheter (PICCO, PV 2015 L20, Pulsion, Germany)) for advanced hemodynamic monitoring. Two central venous catheters were surgically introduced into the internal and external jugular vein for volume administration and injection of cold indicator for transcardiopulmonary thermodilution using a PiCCOplus monitoring system (version 6.0, Pulsion Medical Systems, Munich, Germany). Fluid management was identical for all animals. A basal infusion rate of 13 ml/kgBW/h was administered using hydroxyethyl starch 6% 130/0.4 and Ringer’s solution at a fixed ratio of 1:2. Macrocirculation was assessed continuously and maintained identically in all animals during the entire procedure according to an established algorithm for goal-directed fluid management [20–22]. Body temperature was kept constant between 38 and 39 °C using forced-air warming and a heating pad.

### Surgical procedures and induction of acute pancreatitis

After repositioning of the pigs into supine position, a gastric tube has been placed and the abdomen was opened by a transverse upper laparotomy. A urinary catheter was placed directly into the bladder for urinary drainage. The pancreas and duodenum were mobilized and fixed at the laparotomy incision for intraoperative measurements. After dissection and cannulation of the main pancreatic duct (Vasofix 0.8 mm, B. Braun, Melsungen, Germany) between the pancreas and duodenal wall, a flexible polarographic measuring probe (CCP1, Licox, Kiel, Germany) for continuous measurement of the tissue oxygen tension (tpO2) was placed in the pancreatic head [23, 24]. After a few minutes of equilibration, the baseline values of all parameters (M0) were measured. According to the protocol, the measurements include blood samples, blood gas analysis and measurement of tissue oxygenation (tpO2) and the microcirculation in the pancreatic head with a laser Doppler imager (LDI, Moore, UK). Afterwards, acute necrotizing pancreatitis was introduced by intraductal infusion of glycodeoxycholic acid (GDOC, 10 mmol/l, pH 8, Sigma–Aldrich, St. Louis, MO, USA) over a period of 15 min as previously described, using an automated infusion system (Perfusor® fm (MFC), B Braun, Melsungen, Germany) to avoid pancreatic pressure necrosis [25]. This was paralleled by continuous intravenous infusion of cerulein at 5 μg/kg/h (Sigma–Aldrich, St. Louis, MO, USA). The cannula has been removed, and the pancreatic duct was ligated. Sixty minutes (M1) and 120 min (M2) after completion of the intraductal infusion measurements were repeated. Directly after M2, the animals of group 1 (melatonin treatment) received a bolus of 10 mg/kg melatonin (Sigma–Aldrich, St. Louis, MO, USA) was applied via the central venous catheter. After the start of the therapy, a stabilization period of 30 min was allowed before the effects were measured every 60 min (M3–8). After the last intraoperative measurement (M8), all catheters were removed except the central venous catheter that was subcutaneously tunneled to the dorsal neck of the pig for application of analgesic medication and blood gas testing in the postoperative course. The abdominal cavity and incision of the neck have been closed, and anesthesia was terminated. The animals were extubated, and in case of sufficiently spontaneous breathing, they were transferred to heated boxes in the animal facility. For 7 days, the animals have been closely monitored and analgesics were given every 4–6 h (piritamide 15 mg, equivalent to 10 mg morphine). The animals were kept in small groups of 5–8 animals in a mulched stall in the animal facility with free access to straw and regular diet for pigs. They had a normal light–dark cycle via window. The animals had free access to straw and regular diet for pigs and water from the tap. All animals were checked by a veterinarian after arrival at the animal facility and then daily until death. No intervention was performed prior to the experiment.

Twenty-four hours prior to surgery, they were kept in a separate compartment. After fasting overnight with free access to water, they underwent premedication and anesthesia. Once a day, blood samples and blood gas analysis were performed and the animals were evaluated for their fitness using two scores (fitness and Porcine Well-being (PWB) score) that had been used earlier by our group [26, 27]. Animals surviving the observation period were re-anesthetized on the seventh postoperative day and sacrificed by fast injection of potassium chloride during anesthesia. The pancreas was removed for histopathologic examination and molecular biological analysis. In animals that died during the postoperative course, the pancreas was removed directly after detection of death.

### Histopathological analysis

Representative specimens of the pancreas were taken. Parts of each pancreatic area, that is, head, corpus, and tail were stored in 3.5% buffered formalin, separately. The tissues were then processed and embedded in paraffin, and 5-μm slices were stained with hematoxylin and eosin. The slices were examined by an experienced pathologist. Specimens were examined by a treatment-blinded experienced pathologist. The histopathologic evaluation of the pancreatic lesions was based on a previous publication [28]. Histopathologic changes were evaluated for each pancreatic area, that is, head, corpus, and tail, separately, and for each anatomic region, a total score ranging from 0 (no alterations) to 12 (severe pancreatitis) was determined (Fig. 3). Primary and secondary endpoints are overall survival, histopathological score, and postoperative fitness and well-being score.

### Statistical analysis

Statistical analysis was performed with SPSS® for Windows® (Version 22.0) (SPSS Inc., Chicago, IL). A detectable difference of 25% versus 75% in survival was used to calculate group size. According to the randomization, the treatment group was evaluated versus the control group. Descriptive analysis of the parameters is expressed as median and range due to the low number of subjects per group.

For analysis of the difference between the groups in repeated measurements, the variance analysis for repeated measurements was used to evaluate the change within group, change between groups, and the interaction. The Bonferroni testing was used for post hoc analysis. The survival time was calculated using the log-rank test.

Significance statements refer to *p* values of two-tailed tests that were less than 0.05.

## Results

### Baseline data of animals

The animals were randomized according to the operative procedure into the following two groups: group 1/melatonin group and group 2/non-melatonin group. A total of 18 animals were treated with melatonin, while a total of 20 animals were randomized to the control group. The clinical characteristics of the pigs of the two groups were similar at baseline. In detail, the length was 100 (92–108) cm and weight was 30.7 (25.9–35.5) kg of the pigs of the melatonin group and 98.8 (88–104) cm and 30.3 (26.8–35.1) kg of the pigs of the non-melatonin group (*p* = 0.526 and *p* = 0.745).

### Overall survival of animals

**Fig. 1:**
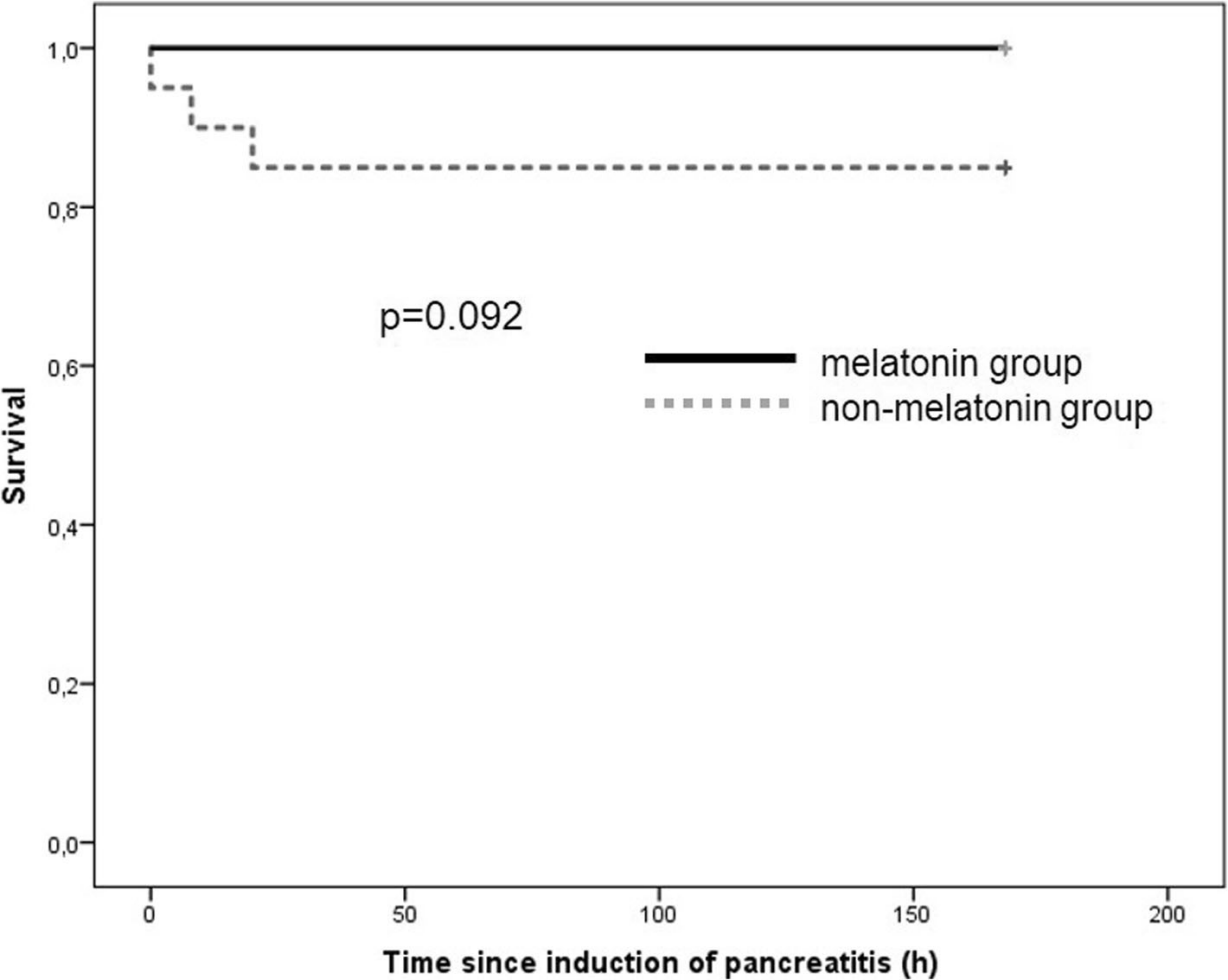
Overall survival

All of the pigs of the melatonin group survived the intra- and postoperative course, whereas three pigs of the control group died. The overall survival rate showed no significant differences (mortality 0% vs. 15%; *p* = 0.23). The survival time was similar in both groups of pigs 168 h (85% CI 168–168) vs. 144 h (95% CI 119–168) (*p* = 0.092; Fig. 1).

### Tissue oxygenation (tp O2 mmHg) of the pancreatic tissue

**Fig. 2:**
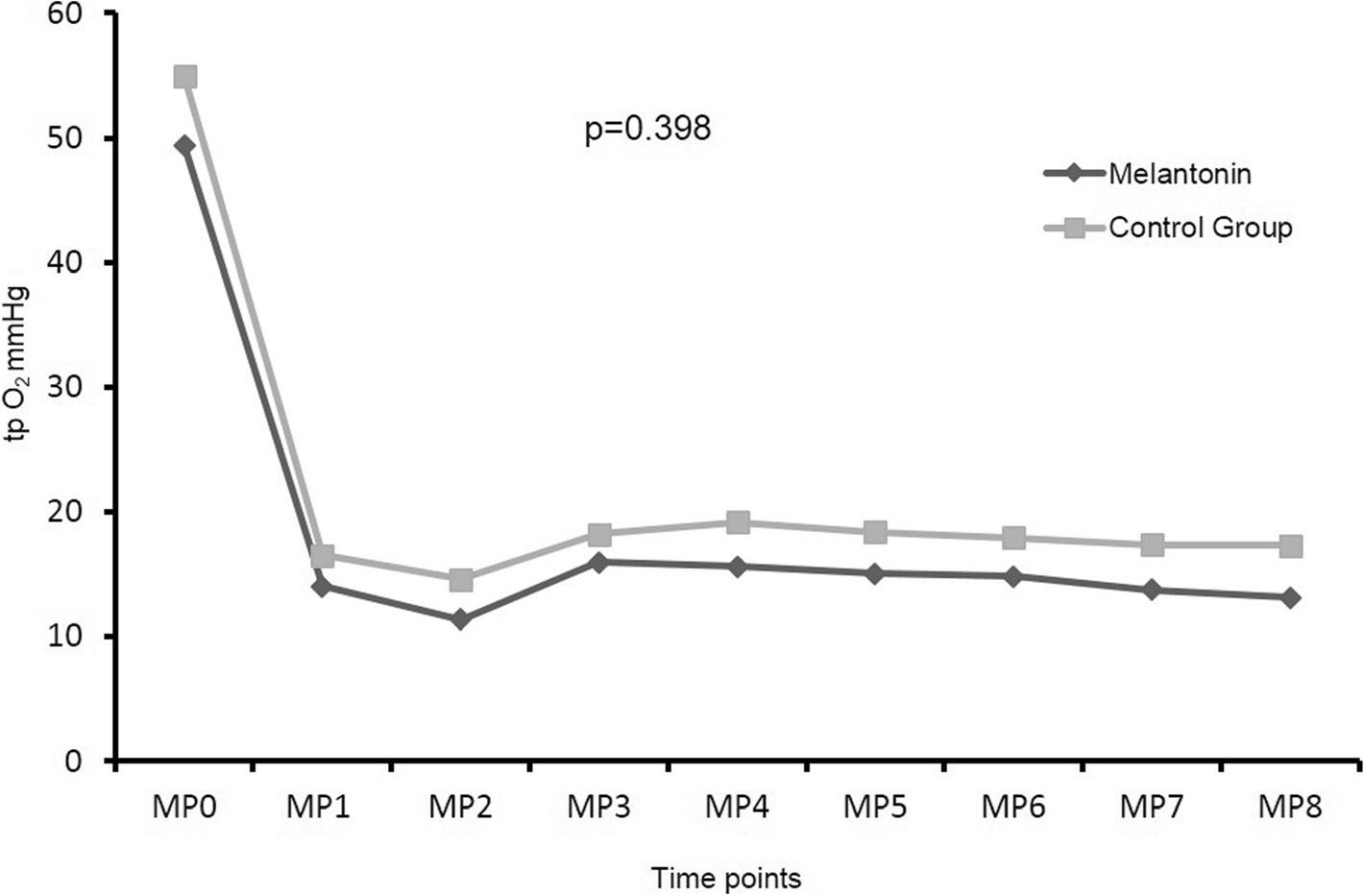
Tissue oxygenation (tp O2 mmHg) of the pancreas

Figure 2 shows the tissue oxygenation of the pancreas during the operative course. No differences were observed regarding the tissue oxygenation of the analyzed two groups. The repeated measurement analysis of variance revealed significant changes within the group (*p* < 0.001) and no significant impact of the group (*p* = 0.398) or the interaction (*p* = 0.844).

### Intraoperative hemodynamic data and chemical blood results

All animals were kept in stable hemodynamic conditions during the operation. Additional files 1 and 2 show the hemodynamic data and the results of the blood tests (Additional files 1 and 2). These data demonstrate that there were no significant differences concerning the analyzed parameters between both analyzed animal cohorts.

### Postoperative fitness and PWB score

As demonstrated in Table 1, pigs of the melatonin group were characterized by a higher fitness and PWB score as compared the pigs of the control group.

**Table 1:** Postoperative fitness and PWB score of the animals

	Postoperative						
	Day 1	Day 2	Day 3	Day 4	Day 5	Day 6	Day 7
Fitness score							
Group 1 (melatonin)	2 (1–4)	3 (2–4)	2 (1–4)	3 (1–4)	3 (1–4)	3 (2–4)	3.5 (2–4)
Group 2 (non-melantonin)	2 (0–3)	2 (0–3)	2 (0–4)	2.5 (0–4)	3 (0–4)	3 (0–4)	3 (0–4)
*p*	*p* = 0.793	*p = 0.034*	*p* = 0.959	*p* = 0.188	*p = 0.013*	*p* = 0.173	*p = 0.003*
PWB score							
Group 1 (melatonin)	31.5 (21.-45)	37 (28–45)	36 (23–48)	41.5 (32–48)	42.5 (35–47)	43 (32–48)	45.5 (33–48)
Group 2 (non-melantonin)	31 (0–39	33.5 (0–41)	33 (0–44)	34 (0–43)	34.5 (0–46)	35 (0–43	33.5 (0–42)
*p*	*p* = 0.689	*p = 0.745*	*p* = 0.256	*p = 0.003*	*p < 0.001*	*p = 0.001*	*p = 0.003*

The repeated measurement analysis of variance revealed significant changes within the group for the postoperative fitness and the PWB score (*p* = 0.001 and *p* < 0.001 respectively). Post hoc analysis revealed significant advantage of the melantonin treatment for both scores (*p* = 0.005 and *p* = 0.003). The interaction was *p* = 0.069 and 0.032 respectively.

### Histopathologic score for severe acute porcine pancreatitis

**Fig. 3:**
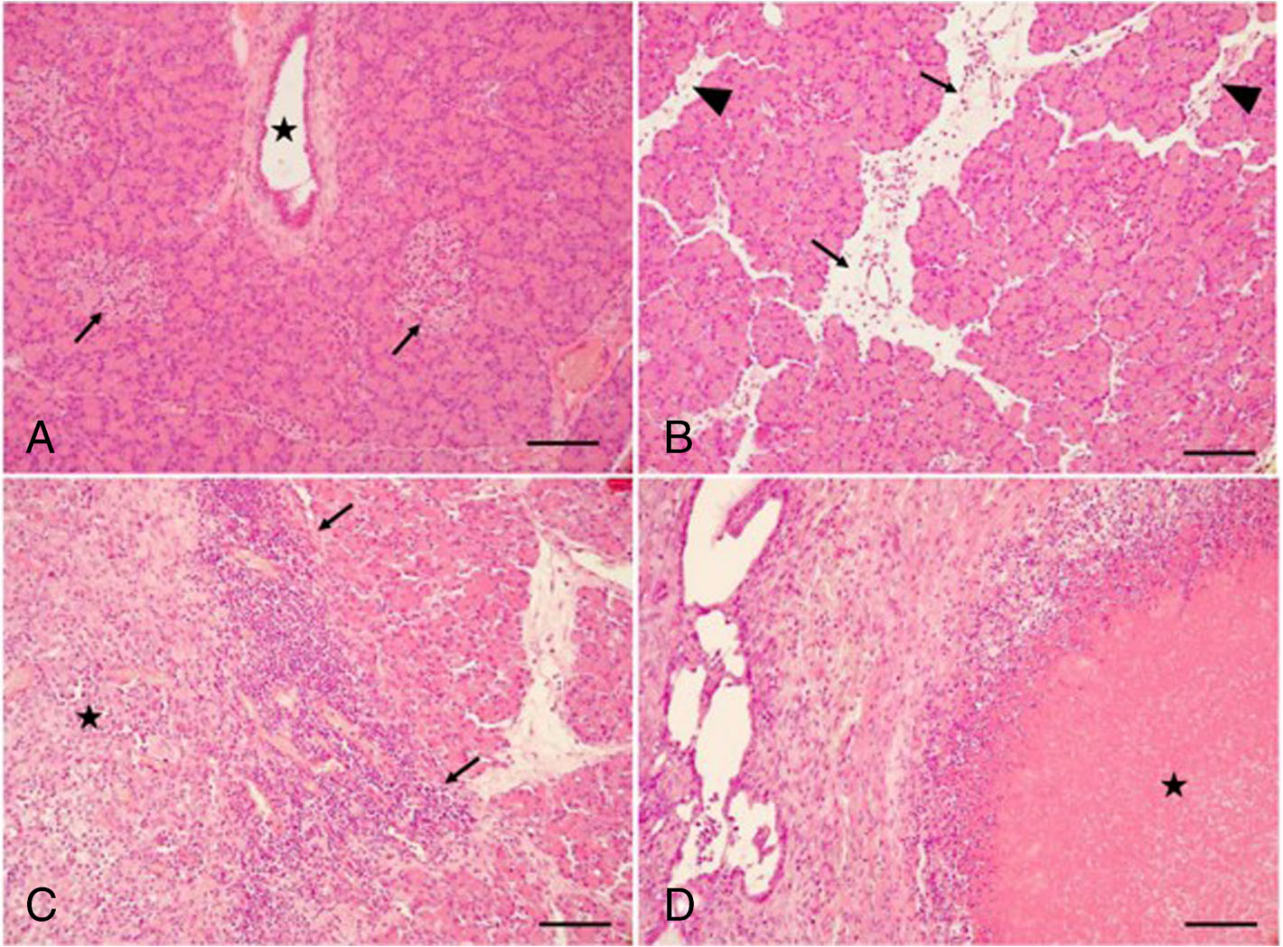
Histological findings in normal and pancreatitis-induced pigs. All tissue sections were stained with hematoxylin–eosin. **a** Normal pancreas showing an intralobular pancreatic duct (star), acini and endocrine islets (arrows). **b** Mild disease with scattered inflammatory cells and interlobular edema (arrows) as well as interacinar edema (arrowheads). In this example, the total histopathological pancreatitis score was 3. **c** Moderate disease with dense inflammatory infiltrates intralobular (arrows) as well as focal tissue necrosis (star). In this example, the total histopathological pancreatitis score was 6. **d** Severe disease with extensive tissue necrosis (star) and dense inflammatory cells around the necrotic area. In this example, the total histopathological pancreatitis score was 11. All bars equal 50 μm

The histopathological analysis revealed that acinar necrosis, fat tissue necrosis, and edema were significantly reduced in the melatonin group as compared to the non-melatonin group (*p* = 0.025, *p* = 0.003, and *p* = 0.028; Table 2). Histological findings in normal pancreas (of pigs that died before randomization) and different stages of the pancreatitis are demonstrated in Fig. 3. All tissue sections were stained with hematoxylin–eosin.

**Table 2:** Histopathologic score for severe acute porcine pancreatitis

	Acinar necrosis	Fatty tissue necrosis	Intralobular inflammation (plasma cells, lymphocytes, and granulocytes outside parenchymal and fatty tissue necrosis)	Edema	Overall
Melatonin group	2.5 (0.0–3.0)	1.2 (0.0–3.0)	2.0 (0.3–3.0)	2.7 (1.0–3.0)	7.9 (3.0–12.0)
Non-melatonin group	2.7 (2.0–3.0)	2.0 (1.0–3.0)	2.5 (1.0–3.0)	3.0 (2.0–3.0)	9.7 (8.3–12.0)
*P*	*p = 0.025*	*p = 0.003*	*p* = 0.150	*p = 0.028*	*p = 0.006*
Score	Acinar necrosis	Fatty tissue necrosis (in relation to plane)	Intralobular Inflammation (plasma cells, lymphocytes, and granulocytes outside parenchymal and fatty tissue necrosis)	Edema	
0	Nil	Nil	Nil	Nil	
1	< 10 acinar cell necrosis/lobule	< 1/3 of plane	Loose infiltrates (≤ 30 cells/HPF)	Interlobular edema	
2	≥ 10 acinar cell necrosis/lobule	≥ 1/3 to < 2/3 of plane	Moderate infiltrates (> 30; ≤ 100 cells/HPF)	Interacinar edema, ≥ 2 lobules	
3	≥ 1/3 of plane	≥ 2/3 of plane	Dense infiltrates (> 100 cells/HPF)	Intercellular edema, ≥ 2 lobules	

Histopathologic pancreatitis total score ranges from 0 (no alterations) to maximal 12 points (severe pancreatitis); a high-power field (HPF) measures 0.3068 mm^2^

*HPF* high-power field

## Discussion

This study was undertaken to analyze the clinical effect of melatonin treatment in an animal model after induced acute pancreatitis. Our study shows that the fitness and PWB score were significantly higher in pigs which were treated with melatonin as compared to the control group. Moreover, melatonin treatment reduced acinar necrosis, fat tissue necrosis, and edema of the pancreatic tissue. Thus, it can be speculated that melatonin might be a useful therapeutic option in the treatment of severe acute pancreatitis.

The initial management of acute pancreatitis includes fluid replacement and optimization of electrolyte balance and providing adequate caloric support [2]. Surgical treatment is necessary in case of acute gallstone pancreatitis or of complications is present which need surgical management [2]. In general, a cholecystectomy is indicated for gallbladder stones or sludge [29]. If only sludge is present, the treatment with ursodeoxycholic acid can be started—as a long-term therapy [29]. An endoscopic biliary sphincterotomy should be performed in patients after cholecystectomy but with still present repeated attacks of pancreatitis with signs of a biliary origin or in patients with sphincter of Oddi dysfunction [29]. Local complications of acute pancreatitis such as pancreatic necrosis, pseudocyst formation, pancreatic duct disruption, and peripancreatic vascular injuries are treated by a combination of endoscopic, radiologic, and surgical techniques [2]. Despite recent advances in diagnostic and therapeutic options of acute pancreatitis, the acute pancreatitis is still associated with high morbidity and mortality [1].

Pathophysiologically, it is believed that trypsinogen activation [30, 31] and inflammatory signaling pathways such as NF-*κ*B signaling induce acinar cell damage and result in acute pancreatitis [32–34]. NF-*κ*B signaling is mediated by pathologic calcium overload and the activation of protein kinase C isoforms [32]. Initially, proinflammatory mediators stimulate I*κ*B kinase resulting in the nuclear location of activated NF-*κ*B [32]. In the nucleus, NF-*κ*B binds to DNA response elements, resulting in the upregulation of proinflammatory cytokine [35]. These proinflammatory mediators, such as tumor necrosis factor-α and interleukin-1, activate the NF-*κ*B signaling pathway in a positive feedback loop [32]. Moreover, the concentrations of cytokines and chemokines causing acinar cell damage increase [32]. In our study, the melatonin treatment reduced the acinar necrosis, fat tissue necrosis, and edema of pancreatic tissue during acute pancreatitis. Thus, it can be speculated that melatonin-induced anti-inflammatory signaling cascades and suppresses the inflammatory pathways. This suggestion is underlined by earlier studies describing melatonin as an activator of antioxidant enzymes including the superoxide dismutase, catalase, glutathione peroxidase, and glutathione reductase [3–5]. Moreover, melatonin has been suggested to reduce radical oxygen and nitrogen species [6–8]. Furthermore, studies have demonstrated that melatonin plays a protective role due to suppression of the gene expression and synthesis of proinflammatory cytokines such as tumor necrosis factor-α and proinflammatory interleukins and prostaglandins [9–12].

Several other antioxidants have been reported in literature, and it has been suggested that increasing the circulating levels of certain antioxidants such as glutathione, n-acetyl-cysteine, α-lipoid acid, vitamin A, vitamin E, and vitamin C helps to prevent the accumulation of free radicals inside our cells thus reducing oxidative stress [36–38]. For example, N-acetylcysteine (NAC), a thiol-containing synthetic compound used in the treatment of acetaminophen toxicity, has been analyzed in experimental hepatic ischemia–reperfusion (I/R) injury which occurs in both liver resection surgery and in transplantation [39]. While some authors described no positive findings [40–42], another study showed that NAC reversed the beneficial effects of ischemic preconditioning [43]. However, our study shows that another antioxidant melatonin reduces inflammatory reaction of pancreatic tissue and enhances fitness score of pigs with acute pancreatitis. Our findings are supported by an earlier study using melatonin in rats, and cells before the pancreatitis were induced [44]. Inositol-requiring 1α (IRE1α)-mediated Jun N-terminal kinase (JNK)/nuclear factor-kappa B (NF-κB) pathway were activated early in AR42J cells and rat AP models [44]. Melatonin significantly inhibited the expression of proinflammatory cytokines and regulated apoptosis-related protein expression [44]. Furthermore, melatonin treatment resulted in reduced pancreatic tissue injury [44]. Thus, the authors suggested that melatonin treatment protects AR42J cells and Sprague–Dawley rats against AP-associated injury, probably through downregulation of IRE1α-mediated JNK/NF-κB pathways [44].

Next, we discuss the right time point of the beginning of the treatment with melatonin after the induction of acute pancreatitis. In our study, we used a short interval between the induction of the pancreatitis and the start of the melatonin treatment, since the direct intraductal injection of bile acid has been shown to induce acute pancreatitis within a few minutes—much faster as compared to gallstone-induced acute pancreatitis [45, 46]. This assumption is underlined by the fact that we macroscopically observed acute severe pancreatitis prior to the beginning of our therapeutic intervention. Moreover, we choose a short interval since the effect of a clinical improvement of the pancreatic microcirculation might be reduced if fulminate necrosis of the pancreas is already present.

The limitations of this study must be noted. Although this is a clinically relevant experimental pig model, the exact mechanism of how the melatonin treatment increased fitness of the animals and changes histopathological results remains elusive. Further functional studies are necessary to fully understand the underlying mechanism of melatonin effect.

Interestingly, we demonstrated that the melatonin treatment increased the fitness and PWB score of the pigs. This observation might be due to the anti-inflammatory effects of melatonin, which in turn might result in enhanced physical performance of the animals.

## Conclusion

The postoperative fitness and PWB score were significantly higher in the pigs of the melatonin group than in the control group. Moreover, melatonin treatment resulted in reduced acinar necrosis, fat tissue necrosis, and edema of pancreatic tissue. Thus, it can be speculated that melatonin might be a useful therapeutic option in acute pancreatitis.

## Additional files

Additional file 1: Intraoperative hemodynamic data of the pigs. (XLSX 13 kb)

Additional file 2: Results of blood test of the animals. (XLSX 12 kb)

## Abbreviations

I/R injury: Ischemia–reperfusion
IL: Interleukin
IRE1α: Inositol-requiring 1α
JNK: Jun N-terminal kinase
NAC: N-acetylcysteine
NF-κB: Nuclear factor-kappa B
PWB: Porcine Well-being
TNFα: Tumor necrosis factor-α
tp O2 mmHg: Tissue oxygenation
